# Sequential Analysis of Livestock Herding Dog and Sheep Interactions

**DOI:** 10.3390/ani10020352

**Published:** 2020-02-22

**Authors:** Jonathan Early, Jessica Aalders, Elizabeth Arnott, Claire Wade, Paul McGreevy

**Affiliations:** 1 Sydney School of Veterinary Science, Faculty of Science, University of Sydney, Sydney, NSW 2006, Australia; jaal7395@uni.sydney.edu.au (J.A.); e_arnott@hotmail.com (E.A.); paul.mcgreevy@sydney.edu.au (P.M.); 2 School of Life and Environmental Sciences, Faculty of Science, University of Sydney, Sydney, NSW 2006, Australia; claire.wade@sydney.edu.au

**Keywords:** livestock, behaviour, herding, sheep, welfare, working dog, livestock herding dog, lag sequential analysis

## Abstract

**Simple Summary:**

Although livestock herding dogs have contributed significantly to Australian agriculture, there are virtually no studies examining the interactions between dog and livestock during herding. One statistical approach that may assist our understanding of such interactions during herding is lag sequential analysis that reveals links between one event and the next. Using high-definition video recordings of herding in a yard-based competition trial and software to code the main dog and sheep behaviours, we identified several significant behavioural interactions. These included the dog ceasing all movement followed by the sheep also ceasing movement; the dog chasing the sheep and a group of sheep escaping the main flock; a single sheep escaping the flock and the dog chasing; sheep initiating movement followed by the dog following; foot-stamping followed by the dog ceasing all movement; and, foot-stamping by the sheep and the dog lip-licking in response. Further statistical analysis found no significant sex differences in the herding styles of the dogs included in the study. Of benefit to livestock herding dog handlers and breeders was the identification of trial score as a predictor of efficient performance.

**Abstract:**

Livestock herding dogs are crucial contributors to Australian agriculture. However, there is a dearth of empirical studies of the behavioural interactions between dog and livestock during herding. A statistical approach that may reveal cause and effect in such interactions is lag sequential analysis. Using 48 video recordings of livestock herding dogs and sheep in a yard trial competition, event-based (time between behaviours is irrelevant) and time-based (time between behaviours is defined) lag sequential analyses identified several significant behavioural interactions (adjusted residuals greater than 2.58; the maximum likelihood-ratio chi-squared statistic for all eight contingency tables identified all sequences as highly significant (*p* < 0.001)). These sequences were: The dog ceasing all movement followed by the sheep also ceasing movement; the dog *chasing* the sheep and a group of sheep escaping the main flock; a single sheep escaping the flock and the dog *chasing*; sheep initiating movement followed by the dog following; *foot-stamping* followed by the dog ceasing all movement; and, *foot-stamping* by the sheep and the dog *lip-licking* in response. Log linear regression identified significant relationships among undesirable behaviours in sheep and both observed trial duration (*p* = 0.001) and trial score (*p* = 0.009). No differences in the herding styles of dogs were identified between sex of dog and frequency of sheep escape behaviours (*p* = 0.355) nor the sex of dog and competition level (*p* = 0.116). The identification of trial score as a predictor of efficient performance confirms the benefits of incorporating extant objective measures to assess livestock herding dogs.

## 1. Introduction

As a component of Australian livestock production, livestock herding dogs are estimated to provide a 5.2-fold return on investment [1]. Their utility, stamina and problem-solving skills are embedded in Australian agricultural folklore. However, the appropriate use of dogs to move livestock and the need to continue to meet public expectations around animal welfare have triggered interest in optimising their deployment. This has also prompted the insertion of clauses referencing the use of dogs in animal welfare standards, both nationally and internationally [2,3].

The work of livestock herding requires a dog with natural instinct combined with well-practiced, stylised movements, often under the command of a handler. In doing so, the dog must, together with the handler, continually adjust to both the livestock and environment. Inadequate training has the potential to compromise the welfare of both dog and livestock [4,5,6,7]. Previous studies have shown the influence of animal handling techniques on both the behaviour and physiology of sheep [8,9]. The fear evident in sheep when dogs penetrate their flight zone is a recurrent theme. Kilgour and De Langen [8] found that, throughout the various activities involving both human and livestock herding dogs, the plasma cortisol concentrations of sheep generally peaked during shearing, followed by dog-mediated herding. The highest individual recorded plasma cortisol concentration in that study occurred when a sheep was bitten by the dog. In a later study, Hemsworth and Coleman [9] found higher cortisol concentrations in sheep when a dog was positioned immediately beside the flock, compared to a dog positioned both within and further than two metres from the flock. Similarly, Beausoleil et al. [10] found that foot-stamping, vigilance and exploratory behaviour of sheep changed in the presence of a dog. Compromised welfare may also affect production outputs, with chronic elevations in plasma cortisol concentrations being associated with poor meat quality and decreased wool production [11,12].

Beyond considerations of sheep welfare, the nature of livestock herding dogs’ responses to ongoing changes in the herding environment and livestock movement determines handlers’ opinions of the dogs’ efficacy. Livestock herding efficiency can be defined as the appropriate balance of movement and pressure that results in the livestock’s timely arrival at their required destination, with minimal ovine (or livestock) distress. It relies on handlers having good dogmanship; that includes the ability to read the dog and respond accordingly [13].

Despite efficiency being a primary requirement of livestock herding dogs, the peer-reviewed literature provides limited information on how effective and efficient movement of livestock can be achieved [14,15,16,17,18,19,20,21,22]. For example, McConnell and Baylis [15] reported observations on visual displays by livestock herding dogs (Border collies) towards sheep during a competition trial and training seminars. While these authors made minor reference to the behaviour of sheep in response to visual displays from each dog, they did not report the sheep’s behavioural responses in their entirety to these dog-initiated behaviours in the study. Siniscalchi et al. [22] identified a significant association between aggressive behaviour and counterclockwise movement amongst a group (n = 15; male n = 6; female n = 9) of herding breeds exposed to sheep for the first time in an enclosed area for six minutes. Aligning with most of the peer-reviewed literature, Burns [14] focused on the valuable behavioural traits of herding dogs when moving livestock, and on how selection for these traits may be incorporated into a breeding program.

To date, analysis of behavioural interactions among dogs and livestock in a herding context has not been undertaken. This may reflect the technical limitations of reliably recording observations in the field and constraints on determining the causes and effects of the two species’ behaviour. Previous attempts to better understand the interactions between livestock and working dogs have focused on the development of an algorithm to capture how livestock herding dogs gather sheep together and what drives the flocking of sheep in the presence of a predator [23,24]. A significant relationship between average time spent immobile and the sex of the livestock herding dogs (Border collies) in open competitions (course format and rules of The International Sheep Dog Society) has also been revealed [25]. Lag sequential methods provide a useful means to assess highly complex behavioural interactions and enable simple investigation of sequential events occurring in observational studies [23,26]. They can be either event-based (where time between behaviours is irrelevant) or time-based (where time between behaviours is defined e.g., within one second). Individual behaviours are defined as either state events (for which duration is measured) or point events (for which duration is irrelevant). Lag sequential analysis relies on the familiarity of behaviours being assessed by the study team and the capacity to reliably identify and code the behaviours being assessed. Technological advancements, such as video editing software, hand-held high definition video cameras and high-capacity portable storage, have significantly lowered the barriers to recording animal behaviour in the field such that the observations can often be conducted post hoc in the comfort of a university research laboratory [13,27]. Lag sequential analysis is a commonly used sequential analysis method, using contingency table analysis, that provides a useful technique to further explore interactions in the herding context. Maximum likelihood-ratio chi squared tests are used to assess dependence between initial and response behaviours while adjusted residuals are used to determine significant behavioural sequences [26,28]. It has the potential to inform improvements, throughout Australia and internationally, in training and performance, welfare and breeding programs.

The current study used video recordings of livestock herding dogs moving sheep during an Australian herding trial competition to identify associations between dog and sheep behaviours and to determine what factors, if any, influence these associations. This involved the novel use of behavioural coding software to assess its suitability for this task. Based on our previous research [1,20,21,29,30,31,32,33] and discussions with Australian working dog experts, we hypothesised that livestock herding dogs with high competition trial scores will move livestock more efficiently than dogs with relatively lower trial scores.

## 2. Materials and Methods

Approval for this study was granted by the University of Sydney Animal Research Ethics Committee (Approval number 15474). The West Wyalong Yard championships, overseen by the New South Wales (NSW) Yard Dog Association, are held annually in western New South Wales, Australia. They feature trial competitions, organised by a local committee, that are solely focused on the yard context. A yard dog trial is defined as: “*a competition in which a dog and its handler must negotiate sheep through a pre-determined course, confined within permanent or temporary sheep yards, within a specified time limit*” [34].

Competition levels classify dogs according to their competition wins and include, in ascending order: *Encourage; Maiden; Novice; Improver*; and, *Open*. *Open* level competitions, unlike the four lower ranked competition levels, do not restrict eligibility. That said, entry into different competition levels is not always restricted to the class of dog referred to, e.g., *Maiden* dogs may enter *Improver* class competitions. Each dog starts each run with 100 points; a judge deducts points based on criteria guided by the competition rules [34].

A total of 48 video recordings of dogs (n = 43; male n = 30; female n = 13; no dogs were neutered) competing in the 2013 West Wyalong Yard championships (held over three days and incorporating the NSW state championship in 2013) were randomly chosen from the *Improver* (n = 15) and *Open* (n = 33) level competitions. These competition levels were selected as they used the same standardised course. As part of the random selection of video recordings of dogs, three dogs (male n = 2; female n = 1) competed in both *Improver* and *Open* competition levels. Video recordings of separate trial runs of two dogs—one in the *Improver* and one in the *Open* level competition were also selected. Two livestock herding dog breeds were represented: the working Kelpie (n = 42) and working Border collie (n = 1). There were 20 handlers of the 43 dogs included in the study (n = 20; male n = 19; female n = 1). The solo female handler had two dogs included in the study. The number of sheep used in each trial run depended on the level of competition: *Improver* (n = 14) and *Open* (n = 16). Approximately 800 Merino sheep were used throughout the three days of competition. In competition trials in Australia, the sheep are commonly sourced from nearby grazing properties and are experienced being moved by livestock herding dogs. The maximum time allowed for a dog to complete the entire yard course was 12 min.

**Figure 1:**
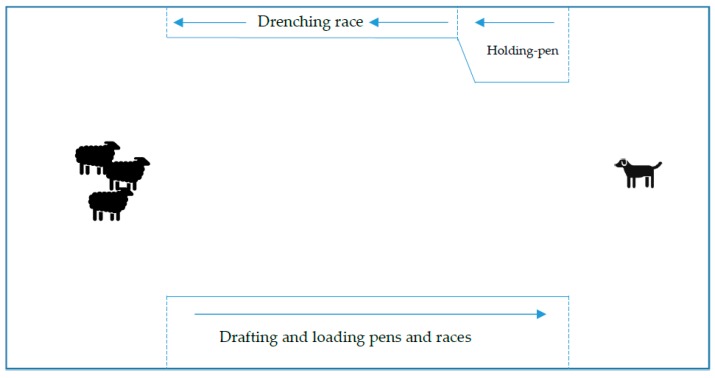
Diagram of competition yard course (not to scale). Illustration of standardised competition yard course. Dog graphic—starting point of dog for competition and video recording coding; Sheep graphic—starting point of sheep for competition and video recording coding; Holding-pen—first holding pen along competition course and end of video recording coding when last sheep entered. Drenching race—commonly used to administer veterinary therapeutics. Solid lines—yard fences; Broken lines—gates. The drafting (commonly used to separate selected sheep from the main flock) and loading pens and races (used to load sheep onto transport vehicles) section of this competition course were excluded from video recording coding. Arrows indicate direction of movement in races.

Video was recorded in high-definition format using a Sony PJ760 high-definition video camera (Sony Corporation, Tokyo, Japan). Behavioural coding was undertaken using specialised observational software [35]. Coding criteria were developed to code the most common sheep and dog behaviours considered relevant to livestock herding. For dogs, movement, discrete types of movement and barking, biting, and lip-licking were determined to be the primary behaviours of interest for this study. For sheep, movement and the ceasing movement and a suite of undesirable behaviours (splitting from the flock, single or a group of sheep escaping from the main flock and foot-stamping) were determined to be the primary behaviours of interest for this study. For each coded video observation, two distinct time-points defined the beginning (the moment the dog initiated its first movement towards the sheep) and end (the moment when all sheep had entered the drenching race holding pen, see Figure 1). The dog was required to cast out from the handler towards the sheep. This marked the beginning of the video recording coding. The dog then needed to drive the sheep towards the drenching race (commonly used in Australia to administer veterinary therapeutics to sheep) holding pen. The entry of the last sheep into the drenching race holding pen marked the end of the video recording coding for this study. The drafting race (commonly used to separate selected sheep from the main flock) and loading pens and race section (used to load sheep onto transport vehicles) of this competition course were excluded from video recording coding due to vision being impaired of the dog and sheep by the yard structures.

The method described below has previously been applied to a lag sequential analysis of dog and human interactions in an obedience training context by our research laboratory at the University of Sydney [13]. To analyse the behavioural interactions between each dog and flock of sheep (and vice versa) in the yarded herding setting, event-based sequential analysis was completed on all recorded observations. Events were divided into state (duration measurable) and point (duration irrelevant) behaviours. Data from this analysis were collated in a contingency table for both dog-sheep behaviours and sheep-dog behaviours. To assess the strength of associations between each dog-sheep behaviour (and vice versa), Yule’s Q was calculated for each sequential behaviour pairing in both contingency tables. Yule’s Q is another method used to assess association between two potentially related events.

A time-based lag sequential analysis was used to explore the relationship between observed behaviours and time. Due to the often fast-paced nature of herding sheep in a yard setting, it was decided to use lags based on one second intervals up to a maximum of three seconds. This generated six contingency tables for analysis. To examine the distribution of chance, a maximum likelihood-ratio chi-squared test was applied to each table. Where the chi-squared value for each table was statistically significant, conditional probabilities (of initial and response behaviours occurring dependently) and unconditional probabilities (of initial and response behaviours occurring independently) were calculated. The adjusted residuals for each sequential pair of behaviours were derived following calculation of the expected frequencies. Adjusted residuals greater than 2.58 were considered to have a strong positive linkage between the paired (initial and response) behaviours [28].

In addition to the method described above, log-linear regression, Spearman’s rank correlations and two-sided correlation analyses were performed to examine the relationships among trial score, competition level, sex (of the dog), latency to complete the coded video observation (referred to as observed trial duration) and sheep behaviours (escape (single and group), foot-stamping, splitting, start and stop).

Statistical analysis was undertaken using Microsoft Excel (Seattle, Washington, USA) except for Spearman’s rank correlations, two-sided correlations and log linear regression analyses which were performed in Genstat 18th edition [36].

The behaviours from the livestock herding dog ethogram and the sheep ethogram used in this study and their associated descriptive statistics are summarised in Table 1.

## 3. Results

Of the 48 video recordings in this analysis, a random subsample of videos (10%) were recoded and the kappa coefficient calculated to assess coding reliability. The kappa coefficient is a form of inter-rater reliability testing that considers agreement occurring by chance. Perfect agreement is given a value of one, whereas chance agreement is given a value of zero [37]. According to Cohen [38], values greater than 0.7 indicate strong agreement. All repeated video observations achieved a kappa statistic of greater than or equal to 0.8.

**Figure 2:**
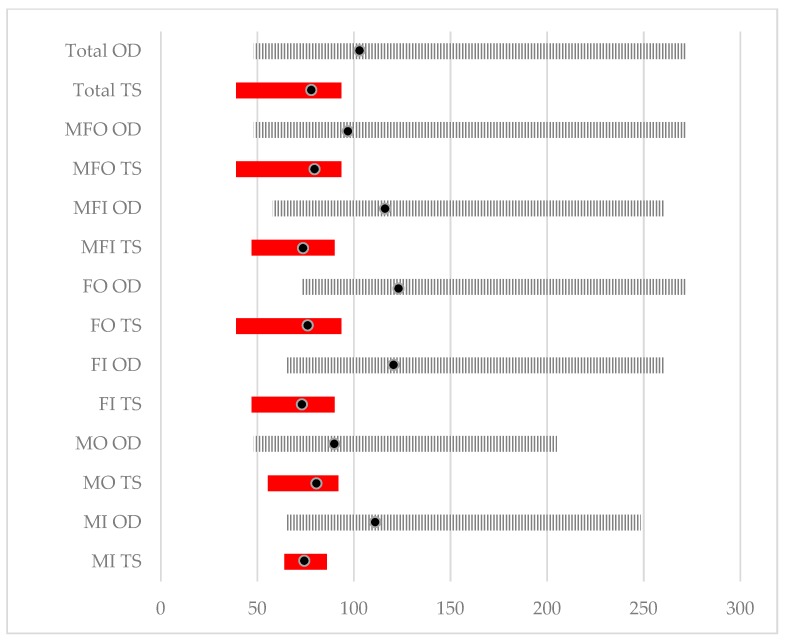
Competition trial results summary: Competition level; Trial score and Observed trial duration. Competition trial scores range and observed trial duration (seconds) range coded divided by sex of dogs and competition level. TS—Trial score range; OD—Observed trial duration range; MI—*Improver* (n = 7); MO—Male *Open* (n = 24); FI—Female *Improver* (n = 7); FO—Female *Open* (n = 6); MFI—Male and Female *Improver* (combined; n = 14); MFO—Male and Female *Open* (combined; n = 32); Circles within ranges indicates the mean value. Competition trial scores range from a perfect score of 100 to 0. Three dogs competed in both *Improver* and *Open* competition levels (male n = 2; female n = 1).

The trial competition results for the 43 dogs included in this study are summarised in Figure 2; see Table A1 in Appendix A for tabulated results. Of the 43 dogs included in this study, three completed the section of the course that was recorded for this study but did not complete the full course and were retired by their handlers, receiving no score.

**Figure 3:**
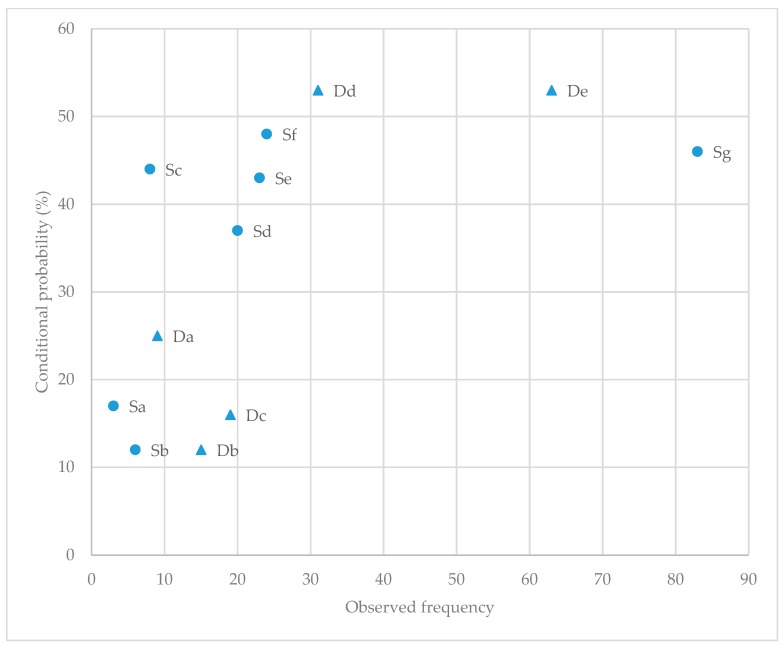
Significant event-based lag sequential analysis results (observed frequencies and conditional probabilities) for sheep behaviour response to dog behaviour and dog behaviour response to sheep behaviour. D—dog-initiated behavioural interactions (denoted by triangle); S—sheep-initiated behavioural interactions (denoted by circle). Da: Chasing—Escape group; Db: Stalking—Foot-stamping; Dc: Moving—Splitting; Dd: Not moving—Stop; De: Moving—Escape single; Sa: Foot-stamping—Lip-licking; Sb: Start—Barking; Sc: Foot-stamping—Not moving; Sd: Stop—Not moving; Se: Stop—Stalking; Sf: Start—Moving; Sg: Escape single and Chasing.

As no recordings of dogs biting sheep were observed, bite was excluded from the analysis. The significant event-based lag sequential analysis results for both dog-initiated and sheep-initiated behaviours are summarised in Figure 3; see Table A2 and Table A3 for complete results. The event-based dog-initiated behaviours revealed five behavioural pairs (*Not moving*—*Stop*; *Moving*—*Splitting*; *Moving*—*Escape single*; *Chasing*—*Escape group*; *Stalking*—*Foot-stamping*) with strong positive linkage. Meanwhile, the sheep-initiated behaviours revealed seven behavioural pairs (*Start*—*Moving*; *Start*—*Barking*; *Stop*—*Not moving*; *Stop*—*Stalking*; *Escape single*—*Chasing*; *Foot-stamping*—*Not moving*; *Foot-stamping*—*Lip-licking*) with strong positive linkages.

**Figure 4:**
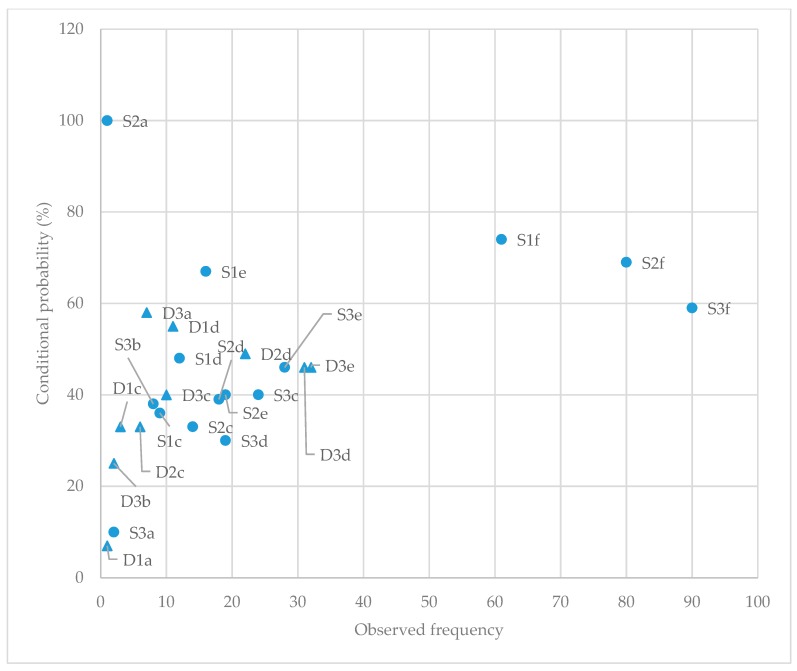
Significant time-based (one, two, three seconds) lag sequential analysis results (observed frequencies and conditional probabilities) for sheep behaviour response to dog behaviour and dog behaviour response to sheep behaviour. D—dog-initiated behavioural interactions (denoted by triangle); S—sheep-initiated behavioural interactions (denoted by circular dot); Number (#) denotes the time-based lag, either 1, 2 or 3 s. D#a: Barking—Splitting; D#b: Crouching—Escape group; D#c: Chasing—Escape group; D#d: Not moving—Stop; D#e: Moving—Escape single; S#a: Foot-stamping—Lip-licking; S#b: Foot-stamping—Not moving; S#c: Stop—Stalking; S#d: Stop—Not moving; S#e: Start—Not moving; S#f: Escape single—Chasing.

The significant time-based lag sequential analysis results for all three time-based lags (one, two and three seconds) are summarised in Figure 4; see Table A4 and Table A5 for complete results. The time-based lag sequential analysis results for dog-initiated behaviours revealed three behavioural pairs (*Not moving*—*Stop*; *Chasing*—*Escape group*; *Barking*—*Splitting*), two (*Not moving*—*Stop*; *Chasing*—*Escape group*), and five (*Moving*—*Escape single*; *Not moving*—*Stop*; *Barking*—*Splitting*; *Crouching*—*Escape group*; *Chasing*—*Escape group*) with strong positive linkage for time-based lags one, two and three seconds respectively. Whereas the results for the sheep initiated behaviours revealed four (*Start*—*Moving*; *Stop*—*Not moving*; *Stop*—*Stalking*; *Escape single*—*Chasing*), five (*Start*—*Moving*; *Stop*—*Not moving*; *Stop*—*Stalking*; *Escape single*—*Chasing*; *Foot-stamping*—*Lip-licking*) and six (*Start*—*Moving*; *Stop*—*Not moving*; *Stop*—*Stalking*; *Escape single*—*Chasing*; *Foot-stamping*—*Not moving*; *Foot-stamping*—*Lip-licking*) behavioural pairs with strong positive linkages for time–based lags one, two and three seconds respectively.

Yule’s Q results for both event-based dog- and sheep-initiated behaviours, including significantly linked associations in bold (adjusted residual greater than 2.58), are summarised in Table A6 and Table A7.

The maximum likelihood-ratio chi-squared statistic, for all eight contingency tables from the event-based and time-based lag sequential analyses are available in Table A8. All sequences were highly significant (*p* < 0.001).

Log linear regression analysis revealed that only trial score and observed trial duration had a significant association with the frequency of sheep behaviours. No significant difference was found between *Improver* and *Open* competition levels (*p* = 0.355). Trial score and the frequency of all sheep behaviours (e.g., *Escape (single and group)*, *Foot-stamping*, *Splitting*, *Start/Stop*) were negatively associated (*p* = 0.009). Analysis of sheep escape behaviour also revealed a significant negative interaction between escape frequency (both *Escape single* and *group*) and trial scores (*p* = 0.04). Observed trial duration correlated significantly with escape frequency (both *Escape single* and *group*) (*p* = 0.001). The association between the sex of the dogs and escape frequency (single and group) was not significant (*p* = 0.355). Additionally, there was no significant sex difference for dogs between *Improver* and *Open* level competition scores (*p* = 0.116).

Spearman’s correlation analysis identified a strong positive association between *Chasing* and *Escape single* attempts in sheep (0.681). *Foot-stamping* in sheep correlated moderately with *Stop* in dogs (0.492). Two-sided test of correlations revealed a strong negative correlation (−0.3, *p* < 0.001) between trial score and observed trial duration confirming that high-scoring dogs were more likely to complete the trial faster than low-scoring dogs.

## 4. Discussion

This is the first study to report dog-sheep interactions using event-based and time-based lag sequential analyses. Plainly, *Escape single* in sheep was the most frequent of all coded behaviours. This behaviour occurred on average over three times per minute, revealing it to be the most common, problematic behaviour that livestock herding dogs contend with in the yard context. This finding identifies *Escape single* as the most important behaviour that a livestock herding dog needs to either prevent or constrain to be an efficient performer. Sheep breaking away alone from the main flock is not exclusive to the yard context and this finding may be of relevance to other contexts, such as mustering (livestock herding in paddocks and along livestock routes). Of the other behaviours measured in this study, *moving* (not including *Stalking* and *Chasing*), closely followed by *Stalking* by dogs and *Start* (all movement of the whole flock advancing in one direction) by sheep, were of the longest duration.

The three undesirable behaviours, *Escape group*, *Splitting* and *Foot-stamping* were much less frequently observed than *Escape single*. This confirms that sheep adopt these behaviours less commonly when confronted with a herding dog. The highest frequency behaviours employed by the livestock herding dogs to group and direct the sheep towards the first pen were *Moving* and *Stalking*.

No instances of dogs *Biting* sheep were recorded during the current study. Due to sheep welfare concerns involving livestock herding dogs, trial competitions in Australia now include rules outlining the appropriate actions by judges when biting is observed, e.g., disqualification when the biting is deemed excessive or inappropriate [34]. The current result concurs with a recent report that most Australian handlers do not consider *Biting* a highly valuable trait in any of the four working and competition contexts: utility, muster, yard and trial [21].

Similar to Payne et al. [13], the current study showed that significant behavioural interactions can emerge up to three seconds after an initiating behaviour. Due to the often fast-paced nature of herding in the yard context and the high frequency of behavioural interactions observed during recording, the current team had anticipated that seemingly paired behaviours occurring beyond three seconds would be of questionable relevance. This may not be the case in the mustering context where livestock herding dogs are able to work sheep often at a slower pace and a greater distance from the handler. So, future studies that explore this prospect may have merit.

Among the dog-initiated event-based behavioural pairings in the current study, the livestock herding dog’s cessation of movement was the most reliable trigger for the sheep to also stop moving. This highlights the need for livestock herding dogs to develop an appropriate balance among their suite of working manoeuvres, to ensure the sheep are moved efficiently to their required destination, while minimising undesirable behaviours. Where the livestock herding dogs lack experience or display excessive excitability, training them to cease all movement on command would have the benefit of minimising undesirable behaviours by sheep.

An unexpected result was the absence of a significant *Moving* and *Start* interaction whereas, in contrast, *Not moving*—*Stop* was significant. The other significant dog-initiated movements were: *Moving*—*Splitting*, *Moving*—*Escape single, Chasing*—*Escape group* and *Stalking*—*Foot-stamping*. That is, of the significant paired interactions resulting from the dog initiating movement, all four ovine responses were undesirable. This confirms the need for livestock herding dogs to show an appropriate balance between stopping and moving sheep in the confined area in yard work that is likely to involve them encroaching on the flight zone of the sheep.

Alternatively, in the case of sheep-initiated movement, the two significant event-based behavioural interactions involved the dog *Moving* and *Barking*. The instances of *Barking* in this behavioural interaction may reflect either the dog’s putative excitement or relief that the sheep had resumed motion. This result may also indicate that, within the yard context, it is the sheep that lead the “dance” with the livestock herding dogs. While this finding appears to counter the intuitive view that it is the dog that is herding the sheep, it is not totally surprising within the yard context. Due to the often-confined nature of sheep yards and resulting close proximity of dogs and sheep during herding, providing the sheep with the opportunity to initiate movement in the intended direction (without being actively forced by the dog) may result in less stressful, more efficient herding. Further research on this behavioural interaction should examine whether this result is confined to the yard context or can be generalized across all working and competition contexts.

The current study identified two significant *Foot-stamping* interactions when *Foot-stamping* occurred first: *Not moving* and *Lip-licking*. This result from the event-based sequential analysis is supported by the moderate positive correlation between *Foot-stamping* in sheep and *Not moving* in dogs. Previous studies [39] have reported a 32% increase in *Foot-stamping* by sheep in the presence of dogs. In sheep, this is an important counter-predator response as it has been shown to be used as a defence mechanism against coyotes and as an appetitive behaviour (initial phase of a behavioural sequence) prior to butting [40]. In the livestock herding context, this has implications for both sheep and dog welfare as well as herding performance. The significant interaction with *Lip-licking*, a displacement sign in dogs [41], indicates that some livestock herding dogs in this study had their confidence challenged by ovine *Foot-stamping*. Livestock herding dog handlers have previously identified the high value of *Motivation and Confidence* and placed high value on and expressed a need for context-specific expression of *Boldness* in their working dogs [21]. The high reported values of these traits may reflect a need for livestock herding dogs to contend with repeated threats by livestock when herding them in close proximity.

Among the time-based lag sequential analysis results for dog-initiated behavioural interactions, paired behaviours that occurred across all three time-based lags were: *Not moving*–*Stop* and *Chasing*–*Escape group*. This finding is consistent with the event behaviour results and was anticipated because the sheep were predicted to either slow or stop once the livestock herding dog had ceased moving. Chasing the sheep in a confined yard environment was expected to penetrate the flock’s flight zone and/or be perceived as highly threatening by the sheep.

Among the sheep-initiated behavioural interactions, four behavioural interactions occurred across all three time-based lags: *Start*—*Moving*; *Stop*—*Not moving*; *Stop*—*Stalking* and *Escape single*—*Chasing*. Again, this finding is consistent with the event-based behavioural interaction results and reflects the expected response by the livestock herding dog. Given that these behavioural interactions occurred over a three second period, this finding indicates that there are instances when the livestock herding dogs may not be immediately aware of each sheep’s behaviour and that there may be a delay before they identify and respond to an undesirable outcome (e.g., sheep escaping or the flock *Splitting*).

Higher trial competition scores for livestock herding dogs were significantly associated with sheep displaying fewer undesirable behaviours and lower observed trial duration. This aligns with the significant inverse relationship between observed trial durations and frequency of escape behaviours by sheep. While these results were expected, this study has confirmed the merit of conducting standardised herding trial competitions as an objective assessment of a livestock herding dog’s performance. Further assessment of livestock herding dogs’ ability to limit undesirable behaviours by sheep should reinforce this outcome.

In contrast to previous studies, the current study revealed no significant difference between male and female livestock herding dogs and competition levels. Sex was also not a significant predictor for escape behaviours (*Escape single* and *Escape group*). While previous studies have identified that male and female dogs behave differently not least as a function of their lifetime exposure to gonadal hormones [42,43] including in herding style [25], further research on the contribution of sex to how dogs’ herd livestock needs to be conducted to more fully interpret those findings.

As the current study is the first to investigate behavioural interactions between dogs and sheep in a working context, several limitations merit consideration. Coding behaviours in two species within a working or competition context requires a significant time commitment. However, depending on the study context, not all behaviours may be displayed. Future use of this approach to measuring behavioural interactions could investigate other working or competition contexts that may identify additional primary behaviours of interest. Additionally, flexibility in coding should be permitted when video recordings identify a behavioural interaction that may not have been expected. Particular attention should be paid to whether a behaviour is coded as a ‘state event’ (where the duration is measured) or a ‘point event’ (where the duration is not measured) as this will have an effect on the interrogation of the data. For some situations, such as herding, where a dog employs different types of movement, future studies should investigate the potential benefit of nesting specific types of movement (e.g., crouching or stalking) into broader movement event states. This may facilitate analysis of specific types of movement as well as all movements combined.

The current study focused on *Improver* and *Open* competition levels because they used the same standardised course. It revealed no significant difference in the interactions of dog and sheep between the two levels of competition. The only practical difference between the competition levels was the minor variation in the number of sheep used, but this was not identified as a significant contributor to any behavioural interactions. This may be due to the likelihood that the minimum number of sheep used in the current competition allowed for consistent flocking behaviour to occur. Coding of different competition course formats or working contexts, using different numbers of sheep, and a larger dataset may reveal differences between the interactions of dog and sheep at various levels of competition or working contexts e.g., mustering in paddocks. Plainly, even though the current study used a standardised course, there is no effective way of completely removing sheep-related variability (e.g., flocks that have learnt the course, flocks without a leader sheep) from studies of this sort. Caution should also be exercised when extrapolating results from yard competitions to working environments beyond the yard context or to other competition formats e.g., three-sheep trials. There are limited differences in the working styles between the working Border collie and the working Kelpie in the Australian yard context: the layout in this yard competition required the dogs to keep close to the sheep to move them through the tight spaces and away from corners. Futures studies using lag sequential analysis in other working environments (e.g., mustering in large, open paddocks) should consider the merit of this method to objectively measure variation in working styles between different herding breeds or even the same herding breed from different geographical locations. Knowledge of the level of training of each dog, training methods used by handlers, and the team’s experience moving sheep in different working contexts may also assist in establishing whether these influence the dogs’ working styles or their behavioural interactions with sheep. These elements are intrinsic to effective application of optimal dogmanship [44].

This study has revealed some potential benefits of using lag sequential analysis to elucidate important interactions between livestock herding dogs and livestock. Exposing these significant behavioural interactions may assist trainers in creating scenarios during training that give dogs the opportunity to repeatedly practice the manoeuvres that are most frequently needed. Additionally, it may help to identify dogs that excel in the most common situations or training scenarios, early on, that can then be developed to remediate weaknesses.

## 5. Conclusions

Video recordings of livestock herding dogs and sheep in a yard trial competition, using event-based and time-based lag sequential analyses, revealed several significant behavioural interactions including the dog ceasing all movement followed by the sheep also ceasing movement, the dog *Chasing* the sheep and a group of sheep escaping the main flock, a single sheep escaping the flock and the dog *Chasing*, sheep initiating movement followed by the dog following, *Foot-stamping* followed by the dog ceasing all movement and *Foot-stamping* by the sheep and the dog *Lip-licking* in response. Log linear regression identified significant relationships among undesirable behaviours in sheep, observed trial duration and eventual trial score. The identification of trial score as a predictor of efficient performance confirms the benefits of incorporating existing objective measures to assess livestock herding dogs. An improved understanding of the frequency and significance of behavioural interactions between livestock herding dogs and livestock should assist handlers in determining effective methods to improve dog performance and dogmanship.

## Figures and Tables

**Table 1:** Ethogram and descriptive statistics summarising the dog and sheep behaviours coded in the current study.

Species	Behaviour	Description	Mean Duration (sec)	Total Duration (sec)	Rate per Minute (mean)	Total Number
Dog	Biting	Grabbing or attempting to grab sheep with mouth	–	–	0	0
	Moving	All movement except stalking and chasing	9.59	1814.05	2.52	199
	Not Moving	Stationary except crouching	4.65	616.22	1.86	139
	Stalking	Moving forward with head and body lowered while staring intently at sheep	7.75	1348.81	2.47	191
	Crouching	Lying down towards or on the ground while stationary	3.94	198.53	1.37	42
	Lip-licking	Tongue actively protrudes from dog’s mouth	–	–	0.59	5
	Chasing	Fast running action toward sheep, following an escape attempt by one or more sheep from the flock	8.05	807.12	1.43	106
	Barking	Vocalisation directed towards sheep	–	–	1.11	31
Sheep	Start	All movement of the whole flock advancing in one direction	24.93	2324.24	1.42	103
	Stop	Stationary, stop moving or flocking in circular motion without advancing	5.71	653.38	1.37	101
	Splitting	Separation of flock in different directions	–	–	1.15	57
	Escape single	Single sheep runs from flock	–	–	3.18	255
	Escape group	Two or more sheep run from flock together	–	–	0.72	23
	Foot-stamping	Foot lifted and placed forcefully on ground while facing towards dog	–	–	1.05	37

“–” non-applicable ‘point event’ for the behaviour where duration was not measured. All other behaviours classed as ‘state events’ where duration was recorded.

## Appendix A

**Table A1:** Competition trial results summary: Competition level; Trial score and Observed trial duration.

	Male		Female		Male and Female		Totals
Competition level—# of videos	Improver n = 7	Open n = 26	Improver n = 8	Open n = 7	Improver n = 15	Open n = 33	Improver and Open n = 48
Trial score range (average)	64–86 * (74.33)	55.5–92 (80.59)	47–90 * (73.07)	39–93.5 (76.07)	47–90 ** (73.65)	39–93.5 (79.63)	39–93.5 ** (77.94)
Observed trial duration coded range (mean) in seconds	64.61–248.33 (111.03)	48.25–205.09 (89.85)	65.21–260.56 (120.51)	73.40–272.32 (123.14)	57.9–260.56 (116.09)	48.25–272.32 (96.91)	48.25–272.32 (102.90)

Competition trial scores and observed trial duration range coded divided by sex of dogs and competition level. Mean values of trial scores and observed trial duration are in parentheses. * One dog retired with no score. ** Two dogs retired with no score.

**Table A2:** Event-based lag sequential analysis results for sheep behaviour response to dog behaviour including observed frequencies, conditional probabilities and adjusted residuals.

Dog Behaviour	Sheep Behaviour					
	Start	Stop	Splitting	Escape S	Escape G	Foot-Stamping
Moving	14:12% (−1.2)	16:14% (−3.1)	**19:16% (3.2)**	**63:53% (3.4)**	5:4% (−0.3)	1:1% (−3.1)
Not moving	10:17% (0.4)	**31:53% (5.8)**	1:2% (−2.2)	11:19% (−3.8)	1:2% (−1.2)	5:8% (0.6)
Stalking	23:19% (1.4)	28:23% (−0.2)	5:4% (−2.4)	52:42% (0.4)	0:0% (−3.1)	**15:12% (3.0)**
Crouching	3:30% (1.3)	3:30% (0.5)	1:10% (0.1)	1:10% (−2.0)	2:20% (2.3)	0:0% (−0.9)
Lip-licking	1:33% (0.9)	0:0% (−1.0)	0:0% (−0.6)	1:33% (−0.3)	0:0% (−0.4)	1:33% (1.9)
Chasing	2:6% (−1.7)	4:11% (−1.8)	6:17% (1.6)	13:36% (−0.6)	**9:25% (6.0)**	2:6% (−0.3)
Barking	1:11% (−0.3)	2:22% (−0.1)	1:11% (0.2)	5:56% (0.9)	0:0% (−0.7)	0:0% (−0.8)

Each paired interaction details three results—Observed frequency: Conditional probability in percentage and adjusted residual in parentheses. Significant positive linkage between behaviours are highlighted in bold. Escape S: Escape single; Escape G: escape group.

**Table A3:** Event-based lag sequential analysis results for dog behaviour response to sheep behaviour including observed frequencies, conditional probabilities and adjusted residuals.

Sheep Behaviour	Dog Behaviour						
	Moving	Not Moving	Stalking	Crouching	Lip-Licking	Chasing	Barking
Start	**24:48% (5.6)**	8:16% (−0.8)	7:14% (−1.6)	3:6% (0.3)	0:0% (−0.8)	2:4% (−4.1)	**6:12% (3.4)**
Stop	6:11% (−1.6)	**20:37% (3.4)**	**23:43% (3.7)**	4:7% (0.8)	0:0% (−0.9)	1:2% (−4.6)	0:0% (−1.6)
Splitting	5:16% (−0.5)	9:28% (1.2)	10:31% (1.2)	4:13% (2.0)	0:0% (−0.6)	4:13% (-2.0)	0:0% (−1.2)
Escape S	24:13% (−2.8)	25:14% (−3.0)	33:18% (−2.1)	7:4% (−1.1)	1:1% (−1.1)	**83:46% (7.8)**	7:4% (0.2)
Escape G	5:28% (1.0)	1:6% (−1.6)	4:22% (−0.1)	0:0% (−1.0)	0:0% (−0.5)	8:44% (1.6)	0:0% (−0.9)
Foot-stamping	3:17% (−0.3)	**8:44% (2.6)**	4:22% (−0.1)	0:0% (−1.0)	**3:17% (6.4)**	0:0% (−2.7)	0:0% (−0.9)

Each paired interaction details three results—Observed frequency: Conditional probability in percentage and adjusted residual in parentheses. Significant positive linkage between behaviours are highlighted in bold. Escape S: Escape single; Escape G: escape group.

**Table A4:** Summary of observed frequencies, conditional probabilities, and adjusted residuals of sheep behaviour in response to dog behaviour across the three time-based lags (one, two and three seconds).

Time Lag (sec)	Dog Behaviour	Sheep Behaviour					
		Start	Stop	Splitting	Escape S	Escape G	Foot-Stamping
1	Moving	7:29% (1.0)	3:13% (−1.2)	5:21% (1.6)	9:38% (0.1)	0:0% (−1.0)	0:0% (−1.3)
1	Not moving	3:15% (−0.9)	**11:55% (4.2)**	1:5% (−1.0)	4:20% (−1.8)	0:0% (−0.9)	1:5% (−0.1)
1	Stalking	8:24% (0.2)	6:18% (−0.6)	0:0% (−2.6)	16:47% (1.5)	0:0% (−1.3)	4:12% (2.1)
1	Crouching	2:33% (0.7)	0:0% (−1.3)	1:17% (0.4)	3:50% (0.7)	0:0% (−0.5)	0:0% (−0.6)
1	Lip-licking	0:0% (−0.5)	0:0% (−0.5)	0:0% (−0.4)	1:100% (1.3)	0:0% (−0.2)	0:0% (−0.2)
1	Chasing	1:11% (−0.8)	0:0% (−1.6)	3:33% (2.1)	2:22% (−1.0)	**3:33% (5.4)**	0:0% (−0.7)
1	Barking	0:0% (−0.5)	0:0% (−0.5)	**1:100% (2.8)**	0:0% (−0.8)	0:0% (−0.2)	0:0% (−0.2)
2	Moving	8:20% (−0.2)	6:15% (−1.5)	9:23% (2.4)	15:38% (0.9)	1:3% (−0.6)	1:3% (−1.5)
2	Not moving	9:20% (−0.2)	**22:49% (4.6)**	2:4% (−1.7)	8:18% (−2.3)	0:0% (−1.6)	4:9% (0.2)
2	Stalking	14:24% (0.7)	13:22% (−0.3)	3:5% (−1.9)	21:36% (1.0)	0:0% (−1.9)	7:12% (1.3)
2	Crouching	3:60% (2.2)	0:0% (−1.3)	1:20% (0.6)	1:20% (−0.6)	0:0% (−0.5)	0:0% (−0.7)
2	Lip-licking	0:0% (-0.7)	0:0% (−0.8)	0:0% (−0.5)	1:50% (0.6)	0:0% (−0.3)	1:50% (2.2)
2	Chasing	2:11% (-1.1)	0:0% (−2.5)	4:22% (1.5)	5:28% (−0.3)	**6:33% (6.6)**	1:6% (−0.4)
2	Barking	0:0% (-1.0)	0:0% (−1.1)	1:25% (0.8)	3:75% (1.9)	0:0% (−0.4)	0:0% (−0.6)
3	Moving	15:21% (-0.3)	8:11% (−2.4)	12:17% (2.0)	**32:46% (2.6)**	1:1% (−1.5)	2:3% (−1.6)
3	Not moving	16:24% (0.7)	**31:46% (5.6)**	1:1% (−2.9)	13:19% (−2.8)	0:0% (−2.2)	7:10% (0.5)
3	Stalking	24:24% (0.9)	21:21% (−0.1)	4:4% (−2.7)	39:39% (1.5)	1:1% (−2.2)	11:11% (1.3)
3	Crouching	3:38% (1.2)	1:13% (−0.6)	1:13% (0.1)	1:13% (−1.3)	**2:25% (2.6)**	0:0% (−0.8)
3	Lip-licking	0:0% (−0.7)	0:0% (−0.7)	0:0% (−0.5)	1:50% (0.5)	0:0% (−0.3)	1:50% (2.3)
3	Chasing	2:8% (−1.7)	0:0% (−2.7)	6:24% (2.1)	5:20% (−1.5)	**10:40% (8.3)**	2:8% (0.1)
3	Barking	1:8% (−1.1)	0:0% (−1.8)	**7:58% (5.3)**	4:33% (0.0)	0:0% (−0.8)	0:0% (−1.0)

Each paired interaction details three results—Observed frequency: Conditional probability in percentage and adjusted residual in parentheses. Significant positive linkage between behaviours are highlighted in bold. Escape S: Escape single; Escape G: escape group.

**Table A5:** Summary of observed frequencies, conditional probabilities, and adjusted residuals of dog behaviour in response to sheep behaviour across the three time-based lags (one, two and three seconds).

Time Lag (sec)	Sheep Behaviour	Dog Behaviour						
		Moving	Not Moving	Stalking	Crouching	Lip-Licking	Chasing	Barking
1	Start	**16:67% (5.2)**	0:0% (−2.0)	4:17% (0.5)	2:8% (2.4)	-	1:4% (−4.5)	1:4% (0.8)
1	Stop	2:8% (−2.1)	**12:48% (6.1)**	**9:36% (3.7)**	0:0% (−0.8)	-	2:8% (−4.2)	0:0% (−0.8)
1	Splitting	3:38% (0.9)	2:25% (1.2)	1:13% (−0.1)	0:0% (−0.4)	-	2:25% (−1.2)	0:0% (−0.4)
1	EscapeS	13:16% (−2.7)	2:2% (−3.9)	3:4% (−3.8)	1:1% (−0.7)	-	**61:74% (7.5)**	2:2% (0.4)
1	EscapeG	1:20% (−0.2)	0:0% (−0.8)	0:0% (−0.9)	0:0% (−0.3)	-	4:80% (1.5)	0:0% (−0.3)
1	Foot-stamping	2:29% (0.3)	2:29% (1.4)	3:43% (2.4)	0:0% (−0.4)	-	0:0% (−2.5)	0:0% (−0.4)
2	Start	**19:40% (3.4)**	5:11% (−0.9)	4:9% (−0.8)	2:4% (0.9)	0:0% (−0.7)	11:28% (−2.7)	4:9% (2.0)
2	Stop	8:16% (−1.0)	**18:39% (5.0)**	**14:33% (4.1)**	3:6% (2.0)	0:0% (−0.7)	2:4% (−5.8)	1:2% (−0.6)
2	Splitting	5:45% (1.8)	3:27% (1.1)	1:9% (−0.3)	0:0% (−0.5)	0:0% (−0.3)	2:18% (−1.7)	0:0% (−0.7)
2	EscapeS	17:14% (−3.0)	6:5% (−4.3)	8:7% (−2.6)	1:1% (−1.6)	1:1% (0.0)	**80:69% (8.0)**	4:3% (−0.3)
2	EscapeG	2:22% (0.0)	0:0% (−1.3)	0:0% (−1.2)	0:0% (−0.5)	0:0% (−0.3)	7:78% (2.2)	0:0% (−0.6)
2	Foot-stamping	4:33% (0.7)	5:40% (2.4)	3:20% (1.2)	0:0% (−0.6)	**1:7% (2.8)**	0:0% (−3.2)	0:0% (−0.7)
3	Start	**28:46% (4.8)**	7:11% (−0.8)	6:10% (−1.9)	2:3% (0.1)	0:0% (−0.8)	14:23% (−2.6)	4:7% (1.8)
3	Stop	11:17% (−1.1)	**19:30% (3.8)**	**25:40% (4.9)**	4:6% (1.7)	0:0% (−0.8)	3:5% (−5.9)	1:2% (−0.8)
3	Splitting	5:29% (0.7)	4:24% (1.0)	2:12% (−0.7)	0:0% (−0.8)	0:0% (−0.4)	6:35% (−0.2)	0:0% (−0.8)
3	EscapeS	24:16% (−2.8)	11:7% (−3.7)	20:13% (−2.3)	2:1% (−1.7)	1:1% (−0.5)	**90:59% (7.6)**	5:3% (0.2)
3	EscapeG	2:15% (−0.6)	0:0% (−1.5)	3:23% (0.5)	0:0% (−0.7)	0:0% (−0.4)	8:62% (1.9)	0:0% (−0.7)
3	Foot-stamping	4:19% (−0.4)	**8:38% (3.1)**	4:19% (0.1)	2:10% (1.8)	**2:10% (4.3)**	1:5% (−3.2)	0:0% (−0.8)

“-” means no data recorded. Each paired interaction details three results—Observed frequency: Conditional probability in percentage and adjusted residual in parentheses. Significant positive linkage between behaviours are highlighted in bold. Escape S: Escape single; Escape G: escape group.

**Table A6:** Yule’s Q for Event-based dog-initiated sequential behaviour paired interactions.

Dog Behaviour	Start	Stop	Splitting	Escape S	Escape G	Foot-Stamping
Moving	−0.20	−0.43	**0.51**	**0.37**	−0.09	−0.85
Not moving	0.08	**0.67**	−0.75	−0.56	−0.53	0.15
Stalking	0.20	−0.03	−0.52	0.05	−1	**0.55**
Crouching	0.43	0.17	0.05	−0.73	0.69	−1
Lip-licking	0.48	−1	−1	−0.16	−1	0.76
Chasing	−0.53	−0.45	0.37	−0.11	**0.86**	−0.11
Barking	−0.18	−0.04	0.11	0.30	−1	−1

Initial behaviours: rows; Response behaviours: columns. Sequences showing significant positive linkage based on adjusted residuals greater than 2.58 are highlighted in bold. Escape S: Escape single; Escape G: escape group.

**Table A7:** Yule’s Q for Event-based sheep-initiated sequential behaviour paired interactions.

Sheep Behaviour	Moving	Not Moving	Stalking	Crouching	Lip-Licking	Chasing	Barking
Start	**0.70**	−0.16	−0.33	0.10	−1	−0.84	**0.70**
Stop	−0.35	**0.48**	**0.51**	0.24	−1	−0.92	−1
Splitting	−0.13	0.24	0.23	0.51	−1	−0.49	−1
Escape S	−0.37	−0.39	−0.27	−0.25	−0.52	**0.80**	0.06
Escape G	0.26	−0.64	−0.02	−1	−1	0.37	−1
Foot-stamping	−0.08	**0.55**	−0.02	−1	**0.97**	−1	−1

Initial behaviours: rows; Response behaviours: columns. Sequences showing significant positive linkage based on adjusted residuals greater than 2.58 are highlighted in bold. Escape S: Escape single; Escape G: escape group.

**Table A8:** Maximum likelihood-ratio chi-squared statistic (*G*^2^) for event-based and each time-based lag sequential analysis for dog-initiated and sheep-initiated behavioural interactions.

Sequence Order	Event	Time 1 sec	Time 2 sec	Time 3 sec
Dog-initiated interactions	118.12	59.82	78.89	135.20
	*p* < 0.001	*p* < 0.001	*p* < 0.001	*p* < 0.001
	*df* = 30	*df* = 30	*df* = 30	*df* = 30
Sheep-initiated interactions	160.22	126.39	134.93	141.28
	*p* < 0.001	*p* < 0.001	*p* < 0.001	*p* < 0.001
	*df* = 30	*df* = 25	*df* = 30	*df* = 30
